# Carbon Dot-Decorated Graphite Carbon Nitride Composites for Enhanced Solid-Phase Microextraction of Chlorobenzenes from Water

**DOI:** 10.3390/nano12030335

**Published:** 2022-01-21

**Authors:** Shengrui Xu, Hailin Liu, Anying Long, Huimin Li, Changpo Chen, Suling Feng, Jing Fan

**Affiliations:** 1 Key Laboratory of Green Chemical Media and Reactions, Ministry of Education, Collaborative Innovation Center of Henan Province for Green Manufacturing of Fine Chemicals, School of Chemistry and Chemical Engineering, Henan Normal University, Xinxiang 453007, China; LiuHailin20211001@163.com (H.L.); ming1391027849@163.com (H.L.); andychen2005@163.com (C.C.); 2 Henan Key Laboratory for Environmental Pollution Control, Key Laboratory for Yellow River and Huai River Water Environmental Pollution and Control, Ministry of Education, School of Environment, Henan Normal University, Xinxiang 453007, China; fanjing@htu.cn; 3 113 Geological Brigade, Guizhou Bureau of Geology and Mineral Resources, Liupanshui 553000, China; longying031042@163.com

**Keywords:** carbon dots, graphite carbon nitride, sorbent, solid-phase microextraction, chlorobenzenes

## Abstract

In this work, carbon dot-decorated graphite carbon nitride composites (CDs/g-C_3_N_4_) were synthesized and innovatively used as a SPME coating for the sensitive determination of chlorobenzenes (CBs) from water samples, coupled with gas chromatography–mass spectrometry. The CDs/g-C_3_N_4_ coating presented superior extraction performance in comparison to pristine g-C_3_N_4_, owing to the enhancement of active groups by CDs. The extraction capacities of as-prepared SPME coatings are higher than those of commercial coatings due to the functions of nitrogen-containing and oxygen-containing group binding, π–π stacking, and hydrophobic interactions. Under optimized conditions, the proposed method exhibits a wide linearity range (0.25–2500 ng L^−1^), extremely low detection of limits (0.002–0.086 ng L^−1^), and excellent precision, with relative standard deviations of 5.3–9.7% for a single fiber and 7.5–12.6% for five fibers. Finally, the proposed method was successfully applied for the analysis of CBs from real river water samples, with spiked recoveries ranging from 73.4 to 109.1%. This study developed a novel and efficient SPME coating material for extracting organic pollutants from environmental samples.

## 1. Introduction

Chlorobenzenes (CBs) are a kind of raw materials for the synthesis of pesticides, dyes, chemical agents, polymers, and other fine chemicals, and have been widely used in modern industries. However, CBs would also endanger human health due to their refractory degradation, high toxicity, fat solubility, and bioaccumulation. CBs are very irritating to the eyes and skin, and can even inhibit the human nerve center and damage the liver and kidneys if water contaminated with CBs is ingested [1,2]. Therefore, CBs have been listed as priority pollutants by many governments all over the world. In view of this, it is essential to develop a simple and sensitive analytical method for monitoring the contamination of CBs in water.

Generally, traditional approaches for the determination of CBs in water have been established by using gas chromatography (GC) or gas chromatography–mass spectrometry (GC-MS) after pretreatment procedures such as liquid extraction and solid-phase extraction [3]. It is well known that the sample pretreatment consumes most of the time and labor during an analytical process. In comparison to traditional pretreatment techniques, solid-phase microextraction (SPME) presented outstanding performances for the extraction of organic compounds from water, owing to its advantages of solvent-free sampling, straightforward operation, and the integration of sampling, extraction, and injection into a single step [4,5]. The extraction capacity for analytes depends on the adsorption performance of coating materials; therefore, SPME coatings play an important role in the sensitive extraction of targeted analytes [6,7]. Although various commercial SPME coatings have been developed, a great effort should be made to further improve the extraction capacity for determining trace analytes.

Up to now, a variety of materials have been investigated as SPME coatings, such as carbon-based materials, metal oxides, metal–organic frameworks, covalent organic frameworks, and polymers, etc. [8,9,10], wherein carbon-based materials displayed glorious extraction performance towards non-polar and slightly polar compounds because of their hydrophobicity and ease of functionalization [11,12,13]. As a type of zero-dimensional carbon-based material, carbon dots (CDs) have been widely used in various fields, such as chemical sensing, biosensing, drug delivery, catalysis, etc. [14,15,16,17], given that CDs possess abundant oxygen-containing groups on the surface, e.g., hydroxyl and carboxyl groups, which provide plentiful active sites for adsorbing targeted compounds through electrostatic attraction, hydrogen bonding, and π–π interactions [18]. However, the application of CDs for extracting targeted compounds from aqueous samples was limited by the isolating difficulty of CDs after adsorption due to their strong hydrophily. Therefore, a supported material can be used for the immobilization of CDs to overcome this drawback [19]. Li et al. [20] synthesized carbon dot@poly(glycidyl methacrylate) (PGMA)@Fe_3_O_4_ nanoparticles as an adsorbent for the solid-phase extraction of polycyclic aromatic hydrocarbons from water samples. The extraction performance was tunable through controlling the doped amounts of CDs and changing the polymer length. Qiu’s group prepared CDs and polyethyleneimine-functionalized CD-decorated silica microparticles as a stationary phase of liquid chromatography [21,22]. Results showed that the decorated CDs enhanced the separation capacities for nucleosides and sulfonamides effectively. Although the adsorption performance of CDs has been investigated by modifications to support materials, alternative supports should be explored for expanding the application of CDs. Furthermore, there are few reports on the research of CDs in SPME. 

Graphite carbon nitride (g-C_3_N_4_) has attracted wide attention as an effective adsorbent for sample pretreatment due to its simple preparation, abundant π-electron structure, hydrophobicity, and excellent thermal and chemical stability [23,24,25,26]. In view of its excellent properties, g-C_3_N_4_ provided great potential as an ideal support for anchoring CDs. In this work, the CD-decorated g-C_3_N_4_ (CDs/g-C_3_N_4_) was synthesized by precipitating CDs into g-C_3_N_4_ sheets in aqueous solution, and was used as a SPME coating for the sensitive determination of CBs in water. The decoration of CDs onto g-C_3_N_4_ exhibited more abundant oxygen- and nitrogen-containing groups and enlarged the surface area, resulting in the improvement of extraction efficiencies for CBs from water. Furthermore, the CDs/g-C_3_N_4_ composites took full advantage of the abundant chemical groups of CDs and the hydrophobicity of g-C_3_N_4_, and overcame the drawbacks of CDs in pollutant-extraction from water due to its strong hydrophily. After optimizing the extraction conditions, the proposed method was finally applied for the analysis of CBs in real river water. To the best of our knowledge, the CDs/g-C_3_N_4_ composites were, for the first time, used for the SPME of pollutants from water. This study not only provided a novel material for SPME coating, but also extended the application field of CDs in SPME techniques.

## 2. Experimental

### 2.1. Reagents and Materials

The CBs standards, including 1,3,5-trichloro-benzene (1,3,5-TCB), 1,2,4-trichloro-benzene (1,2,4-TCB), 1,2,3-trichloro-benzene (1,2,3-TCB), 1,2,3,5-tetrachloro-benzene (1,2,3,5-TCB), 1,2,4,5-tetrachloro-benzene (1,2,4,5-TCB), pentachloro-benzene (PCB), hexachloro-benzene (HCB), and pentachloronitro-benzene (PCNB) were purchased from AccuStandard (New Haven, CT, USA). Melamine was purchased from Damao Chemical Reagent Factory (Tianjin, China). Ethanol, ethylenediamine, and citric acid were obtained from China National Pharmaceutical Group Co., Ltd. (Beijing, China). Stainless steel wire (O.D. 100 μm) was produced by Hubei Baofeng Industrial Co., Ltd. (Shenzhen, China). Sylard184 silicone elastomer was purchased from Dow Silicones Corporation (Seneffe, Belgium). Commercial SPME coatings, including PA, DVB, and PDMS were provided by ANPEL Laboratory Technologies Inc. (Shanghai, China).

### 2.2. Apparatus

The microstructures of the CDs/g-C_3_N_4_ composites were characterized by a scanning electron microscope (SEM, SU8010, Hitachi, Tokyo, Japan) and a high-resolution transmission electron microscope (HRTEM, JEM-2100, JEOL, Tokyo, Japan). X-ray diffraction spectra (XRD, X’Pert3 Powder, PANalytical B.V., Almelo, Holland) was used to characterize the compositions of as-prepared materials. Fourier-transform infrared (FTIR) spectra were recorded by a Thermo Nicolet NEXUS spectrometer (Thermo Fisher Scientific, Waltham, MA, USA) in the range of 4000–400 cm^−1^. The surface chemical groups were detected by X-ray photoelectron spectroscopy (XPS, VG Multilab 2000 X spectrometer, Thermo Fisher Scientific, Waltham, MA, USA). The BET surface area and pore size distribution of CDs/g-C_3_N_4_ composites were obtained by a nitrogen adsorption/desorption apparatus (Micromeritics ASAP 2020 M, Atlanta, GA, USA). Thermogravimetric analysis was carried out on a comprehensive thermal analyzer (TG, STA449C, Netzsch, Selb, Germany). CBs were determined using a 7890B–7000D GC-MS instrument (Agilent, Santa Clara, CA, USA) equipped with a HP-5 MS fused quartz capillary column (30 m × 0.25 mm × 0.25 μm).

### 2.3. Synthesis of CDs/g-C_3_N_4_ Composites

The preparation of the CDs/g-C_3_N_4_ composites was carried out by simple precipitation of CDs on g-C_3_N_4_ in aqueous solution. The CDs were synthesized using a hydrothermal process, according to the literature, with modifications [27]. Typically, 5 g citric acid and 1.5 mL ethylenediamine were dissolved in deionized water (50 mL). Then, the solution was placed into a 100-mL Teflon-lined autoclave and heated at 180 °C for 5 h. After cooling to room temperature, the CDs were obtained, followed by dialysis for 2 days using a dialysis membrane with a molecular weight cut-off of 3500 daltons (Viskase, Lombard, IL, USA). 

The g-C_3_N_4_ was prepared through a facile calcination procedure in a muffle furnace according to previous reports [28]. A total of 10 g melamine powder was placed into a crucible with a cover, and then transferred to a muffle furnace for annealing for 4 h at 550 °C with ramp rate of 5 °C min^−1^. The g-C_3_N_4_ was obtained after cooling down to room temperature. For the preparation of CDs/g-C_3_N_4_ composites, 500 mg g-C_3_N_4_ powder was added into a CD aqueous solution, followed by stirring thoroughly for 4 h at room temperature. Then, the CDs/g-C_3_N_4_ composites were observed after centrifugation, washing, and drying in a vacuum oven. The doping amounts of CDs on g-C_3_N_4_ were controlled by changing the CDs concentration in solution (1, 5, 10, and 20 mg mL^−1^), which were denoted as CDs/g-C_3_N_4_-1, CDs/g-C_3_N_4_-2, CDs/g-C_3_N_4_-3, and CDs/g-C_3_N_4_-4, respectively.

### 2.4. Fabrication of SPME Fiber Based on CDs/g-C_3_N_4_ Composites

The fabrication of the SPME coating based on the CDs/g-C_3_N_4_ composites was carried out by physical adhesion with silicone glue, according to reported literatures [29,30,31]. In a typical procedure, a stainless-steel wire with a length of 3–4 cm was washed thoroughly by acetone, hydrochloric acid (3 mol L^−1^), and deionized water, respectively. The cleaned stainless-steel wire was immersed into silicone sealant solution, which consisted of PDMS pre-polymer and a curing agent with mass ratio of 10:1. Then, the stainless-steel wire was dipped into CDs/g-C_3_N_4_ composites powder to form a uniform coating after curing at 120 °C in an oven. Finally, the SPME fiber based on CDs/g-C_3_N_4_ composites was fabricated by assembling stainless-steel wire with an empty SPME needle, and was aged for 20 min at 250 °C in a GC injector before the extraction procedures.

### 2.5. SPME Procedure and Real Samples Analysis

The SPME processes were carried out in 20-mL commercial vials containing 10 mL aqueous solution with an immersion mode. In detail, the SPME fiber was immersed into aqueous solution for extraction for 10–50 min at temperatures of 30–70 °C. After extraction, the SPME fiber was inserted into the GC-MS inlet for desorption. The operating parameters of the GC-MS are shown in Section S1 and Table S1. The real water samples, numbered 1#, 2#, and 3#, were collected from the river located in Puding County, Guizhou Province. After collection, the samples were stored in a refrigerator at 4 °C before analysis by the proposed method.

## 3. Results and Discussion

### 3.1. Characterizations of CDs/g-C_3_N_4_ Composites

The microtopography, structural compositions, chemical groups, specific surface area, and pore size distribution of materials can reveal the formation of microstructures and the active adsorption sites of composites. Therefore, various characterizations were carried out to investigate the properties of CDs/g-C_3_N_4_ composites, including SEM, TEM analysis for microtopography, XRD, FTIR, and XPS analysis for crystal structures and chemical group compositions, and N_2_ adsorption–desorption and pore size distribution isotherms for surface area and porosity. 

**Figure 1:**
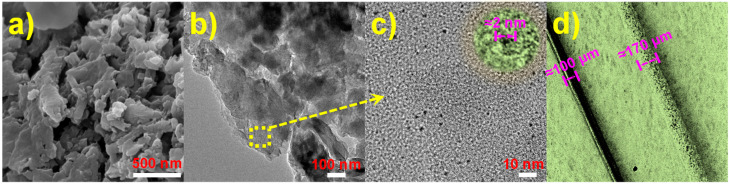
SEM (**a**) and TEM (**b**,**c**) images of CDs/g-C_3_N_4_ composites, magnified TEM image (**c** (inset)) of CDs anchored on composites, and optical microscope image of as-prepared SPME fiber (**d**).

The SEM and TEM were used for investigating the microtopographies of CDs/g-C_3_N_4_ composites (Figure 1). As shown in Figure 1a, as-prepared CDs/g-C_3_N_4_ composites present the irregular stacked structure composed of lamellae. TEM images (Figure 1b,c) show that as-prepared CDs display a circular structure with diameters of approximately 2 nm (Figure 1c (inset)), and the CDs are uniformly distributed on the surface of the g-C_3_N_4_ sheets without visible aggregation. The interactions of CDs and g-C_3_N_4_ can be ascribed to π–π stacking [32]. The optical microscope image (Figure 1d) shows that the uniform coating is formed on the surface of the stainless-steel wire, and the coating thickness is calculated to be approximately 35 μm.

**Figure 2:**
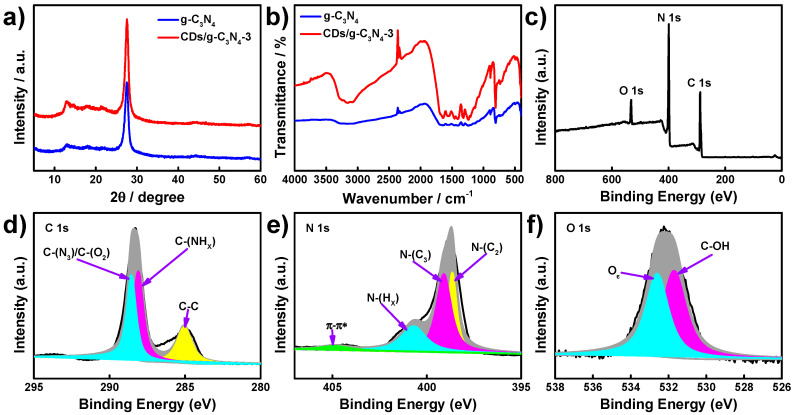
XRD patterns (**a**) and FTIR spectra (**b**) of g-C_3_N_4_ and CDs/g-C_3_N_4_, XPS survey spectrum (**c**), and high-resolution XPS spectra of C 1 s (**d**), N 1 s (**e**), and O1 s (**f**) for CDs/g-C_3_N_4_.

To investigate the crystal structures of CDs/g-C_3_N_4_ composites, XRD patterns were recorded for pristine g-C_3_N_4_ and CDs/g-C_3_N_4_-3 (Figure 2a). It can be seen that the XRD patterns display two peaks at 13.1° and 27.6°, which can be ascribed to the planes of (100) and (002), respectively, referring to g-C_3_N_4_ (JCPDS 87-1526) [33]. The stronger peak, at 27.6°, is derived from inter-planar stacking of the aromatic system, and the diffraction peak at 13.1° describes the formation of in-plane repeated tri-s-triazine units [34,35]. There is no diffraction peak recorded that corresponds to CDs, due to their low contents on the composites, and the doping of CDs does not change the crystal structure of g-C_3_N_4_. 

Figure 2b exhibits the FTIR spectra of pristine g-C_3_N_4_ and CDs/g-C_3_N_4_ composites. Multiple peaks from 1200 to 1700 cm^−1^, which formed both on g-C_3_N_4_ and CDs/g-C_3_N_4_ composites, correspond to the stretching vibration of heptazine-derived repeating units and C–N or C–NH–C groups. The peaks at 806.1 cm^−1^ reflect the flexural vibrations of triazine units, and the wide peak that emerges at 3000–3500 cm^−1^ is ascribed to NH_2_ or NH groups [33,36]. In comparison with g-C_3_N_4_ and CDs/g-C_3_N_4_ composites, the obtained FTIR spectra are similar in shape, which indicates that the decoration of CDs does not change the chemical structures of g-C_3_N_4_. It should be noted that the adsorption peaks are enhanced after the CD-decoration, with the result that more abundant chemical groups are formed.

XPS analysis was performed to determine the chemical composition and surface groups of CDs/g-C_3_N_4_ composites. As shown in the survey scan spectrum (Figure 2c), three peaks are observed at 288.3, 398.8, and 532.2 eV for CDs/g-C_3_N_4_, which are attributed to C 1 s, N 1 s, and O 1 s, with atom contents of 47.17%, 46.62%, and 6.21%, respectively. The high-resolution spectrum of C 1 s (Figure 2d) can be deconvoluted into three peaks at 284.9, 288.1, and 288.6 eV, which are attributed to C-C, C-(NH_X_), and C-(N_3_), respectively [37]. The N 1 s spectrum (Figure 2e) is deconvoluted into four peaks at 398.6, 399.1, 400.9, and 404.8 eV corresponding to the triazine ring of nitrogen (N-(C_2_)), tertiary nitrogen atoms (N-(C_3_)), N-H_X_, and π–π* stacking [38]. The O 1 s spectrum (Figure 2f) can be divided into two peaks at 531.7 and 532.6 eV, which can be ascribed to the C-OH group and adsorbed O on the surface [39]. The abundant chemical groups formed on CDs/g-C_3_N_4_ composites can provide huge active adsorption sites for analytes.

**Figure 3:**
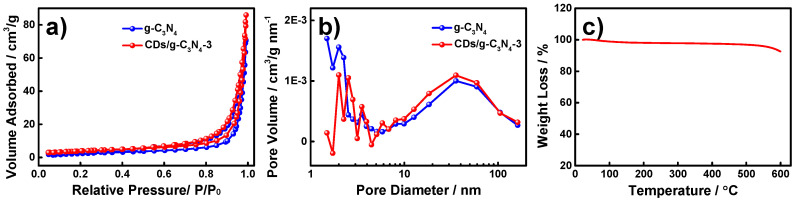
N_2_ adsorption–desorption isotherms (**a**) and pore diameter distributions (**b**) of g-C_3_N_4_ and CDs/g-C_3_N_4_, TG curve of CDs/g-C_3_N_4_ (**c**).

Figure 3a,b show the N_2_ adsorption–desorption and pore size distribution isotherms of pristine g-C_3_N_4_ and CDs/g-C_3_N_4_ composites. The BET specific surface area of CDs/g-C_3_N_4_ was calculated to be 20.67 m^2^ g^−1^, which was higher than that of pristine g-C_3_N_4_ (13.34 m^2^ g^−1^). The average pore diameters of pristine g-C_3_N_4_ and CDs/g-C_3_N_4_ were obtained by the BJH method with values of 33.7 and 39.3 nm, respectively, indicating the formation of mesoporous structures. More details referring to the BET and BJH results are listed in Table S2. A higher specific surface area and mesoporous structures improved the adsorption sites and analyte diffusion [40]. Furthermore, the thermal stability of as-prepared materials was also evaluated by TG analysis, because that SPME fiber needs to be inserted into the GC-MS inlet for desorption at 260 °C. The TG curve (Figure 3c) demonstrates that as-prepared CDs/g-C_3_N_4_ composites have no obvious weight loss within 500 °C, indicating that the composites possess excellent thermal stability.

### 3.2. SPME Performance of CDs/g-C_3_N_4_ Composites

**Figure 4:**
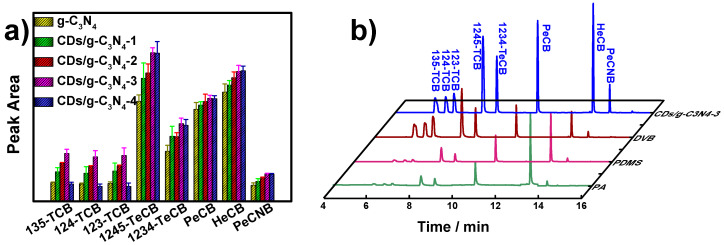
Extraction efficiencies of pristine g-C_3_N_4_ and CDs/g-C_3_N_4_ composites with different doping amounts of CDs (**a**); GC-MS chromatograms of CBs by CDs/g-C_3_N_4_-3 and commercial SPME coatings (**b**). Error bars represent the standard deviation of the mean (*n* = 3).

The extraction performances of pristine g-C_3_N_4_ and CDs/g-C_3_N_4_ coatings with various doped-CD amounts were investigated. As shown in Figure 4a, the extraction efficiencies of CDs/g-C_3_N_4_ display a distinct improvement towards eight CBs, in comparison to pristine g-C_3_N_4_. The extraction efficiencies increase gradually with the enlarged amounts of CD-doping, and the maximum extraction capacity are achieved with CDs/g-C_3_N_4_-3. The further enlargement of CD amounts lead to a diminishment in extraction efficiencies, especially for 135-TCB, 124-TCB, and 123-TCB. Furthermore, the comparisons of extraction capacities for CBs with commercial SPME coatings were also studied to verify the advantages of the CDs/g-C_3_N_4_ coating (Figure 4b). Results show that the CDs/g-C_3_N_4_ coating presents superior performance, compared to the PA, PDMS, and PDMS/CAR/DVB coating. The enhancement in extraction capacities for CDs/g-C_3_N_4_ coating can be ascribed to the following reasons. Firstly, the adsorption of CBs on CDs/g-C_3_N_4_ composites mainly depends on the chemical group binding, π–π stacking, and hydrophobic interactions. Secondly, abundant nitrogen-containing and oxygen-containing groups on CDs/g-C_3_N_4_ are beneficial for bonding with compounds containing polar groups. The addition of CDs introduced more polar groups for composites. However, excessive CD amounts also decrease the hydrophobic interactions between analytes and materials, especially for trichlorobenzenes.

### 3.3. Optimization of SPME Conditions

To further improve the extraction efficiencies towards CBs, the SPME conditions, including extraction temperature, extraction time, salt contents, and pH, were optimized with spiked aqueous solutions with a concentration of 5 ng mL^−1^.

**Figure 5:**
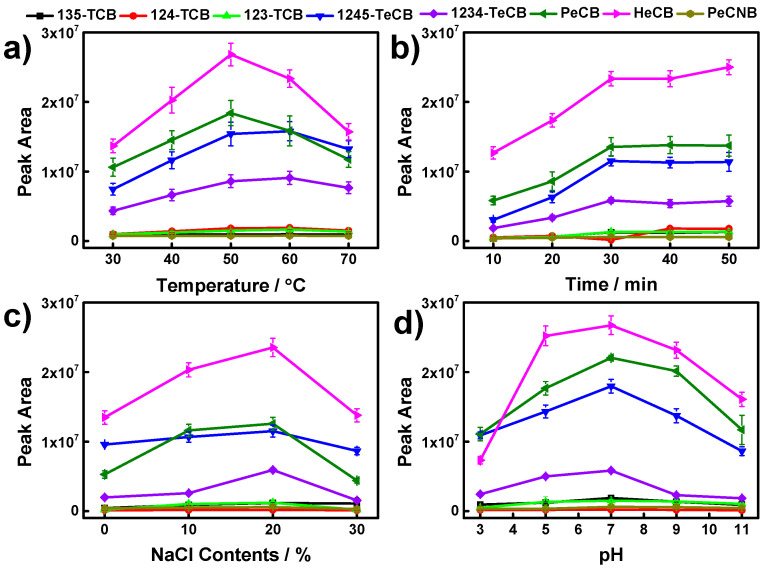
Effect of extraction temperature (**a**), extraction time (**b**), salt concentration (**c**), and pH (**d**) on the extraction efficiencies of the CDs/g-C_3_N_4_ SPME coating for CBs. Error bars represent the standard deviation of the mean (*n* = 3).

Extraction temperature plays an important role on the extraction efficiency for SPME. In general, increased temperature can facilitate the diffusion rate of analytes, and thus, enables the extraction process to reach equilibrium in a short time. However, excessive temperature will decrease the distribution coefficient between the sample matrix and the fiber coating for analytes, which lead to a severe diminishment of the extraction. Therefore, the extraction efficiencies towards CBs for the CDs/g-C_3_N_4_ coating were evaluated in the temperature range of 30–70 °C. It can be seen from Figure 5a that the extraction efficiencies towards eight CBs exhibit a trend of increasing before decreasing with elevating temperature. The maximum extraction efficiencies are observed at an extraction temperature of 50 °C.

Enough extraction time is indispensable to achieve the optimum extraction efficiency, due to the fact that SPME is an equilibrium-based process. Herein, the effect of extraction times ranging from 10 to 50 min on the extraction efficiencies for eight CBs was investigated. As shown in Figure 5b, the extraction efficiencies towards CBs increase, following the increase of extraction time from 10 to 30 min. With further prolonged extraction time, the extraction efficiencies maintain a constant value, which indicates that the SPME process reaches equilibrium.

A certain salt concentration in aqueous solution can effectively decrease the analyte solubility, which leads to an increase of extraction efficiency for non-polar and weak polar compounds [41]. In this work, NaCl was used for adjusting the salt concentration in aqueous solution. The extraction efficiencies of as-prepared SPME coating towards eight CBs were studied in aqueous solution with NaCl contents varying from 0 to 30% (Figure 5c). Results show that the extraction efficiencies display a distinct increase with an increase of NaCl content from 0 to 20%. However, further increased NaCl content leads to a certain reduction in extraction efficiencies, which can be ascribed to excessive NaCl adhesion on the surface of the coating during the extraction process, resulting in the inhibition of active adsorption sites [29,42].

The extraction efficiencies can also be affected by solution pH due to changes in the surface charge of coating materials and analytes [43,44]. The effect of solution pH ranging from 3 to 11 on the extraction efficiencies of as-prepared SPME coatings are evaluated herein (Figure 5d). It can be observed that the highest extraction efficiencies were obtained with solution pH of 7. Therefore, the solution pH was set to 7 for further experiments.

### 3.4. Method Performance

Under the optimized conditions, the parameters for method-performance evaluation, such as linearity, relative standard deviations (RSDs), limits of detection (LODs), and limits of quantitation (LOQs), are summarized in Table 1. Results show that the proposed method exhibits excellent linearity for analyzing eight CBs in a concentration range of 0.25–2500 ng L^−1^, with a linear coefficient (R^2^) of 0.9822–0.9996. The LODs and LOQs were calculated to be 0.002–0.086 ng L^−1^ and 0.007–0.288 ng L^−1^, according to three and ten times of signal-to-noise, respectively. Method precision was evaluated by the values of RSD. As can be seen from Table 1, the obtained RSDs for one single fiber range from 5.3 to 9.7%, and the RSDs among five SPME fibers are in the range of 7.5–12.6% towards eight CBs. Furthermore, comparisons with other methods that have been reported in the literature for the determination of CBs from water were investigated to verify the advantages of the proposed method. As shown in Table 2, the proposed method presents a higher sensitivity for the determination of CBs in water by comparing with other reported methods in terms of LODs and LOQs. Excellent linearity, high sensitivity, and good reproducibility enable the proposed method to be of great potential in real water analysis. Furthermore, the lifetime of as-prepared SPME fiber based on CDs/g-C_3_N_4_ composites was also evaluated in this study. It can be seen from Figure S1 that no significant loss occurred in the extraction efficiency of as-prepared SPME fiber for eight CBs after 120 repeated uses.

### 3.5. Real Water Samples Analysis

The proposed method was finally used to determine CBs from real water samples (1#, 2#, 3#), which were collected from the river located in Puding County, Guizhou Province. The analysis results for the three collected water samples are listed in Table 3. It was found that no CBs were detected in the sample 1#; however, 135-TCB and 123-TCB were detected in samples 2# and 3#, which were collected from a location that is near to a port. Recoveries were obtained, ranging from 73.4 to 109.1%, for eight CBs from water samples with spiked concentrations of 10 ng L^−1^. Satisfactory recoveries illustrate the applicability of the proposed method for the analysis of trace CBs in real water samples.

## 4. Conclusions

In conclusion, CDs/g-C_3_N_4_ composites were synthesized and innovatively applied as SPME coatings for the sensitive determination of eight CBs from water coupled to GC-MS. The doping of CDs improved the extraction efficiency, owing to their abundant chemical groups, and enabled CDs/g-C_3_N_4_ composites exhibit superior extraction efficiency in comparison to commercial coatings. The proposed method presented extremely low LODs, ranging from 0.002 to 0.086 ng L^−1^ for CBs, which is better than the methods reported in the literature. Finally, the proposed method was used for the analysis of real river waters successfully. Outstanding properties, such as high sensitivity and excellent reproducibility, gives the proposed method great potential for the analysis of trace pollutants from aqueous samples.

## Figures and Tables

**Table 1:** Analytical performance of the CDs/g-C_3_N_4_ SPME coating towards CBs.

Analytes	Linear Ranges (ng L^−1^)	R^2^	LODs (ng L^−1^)	LOQs (ng L^−1^)	RSDs (%)	
					One Fiber (*n* = 7)	Fiber-to-Fiber (*n* = 5)
135-TCB	0.25–2500	0.9989	0.003	0.010	8.0	9.1
124-TCB	0.25–2500	0.9974	0.086	0.288	9.7	12.6
123-TCB	0.25–2500	0.9948	0.033	0.108	9.4	10.3
1245-TeCB	0.25–2500	0.9865	0.007	0.024	5.3	7.5
1234-TeCB	0.25–2500	0.9822	0.016	0.053	7.7	9.5
PeCB	0.25–2500	0.9933	0.005	0.016	8.5	8.9
HeCB	0.25–2500	0.9996	0.002	0.007	6.1	8.2
PeCNB	0.25–2500	0.9945	0.024	0.083	7.2	9.8

**Table 2:** Comparisons with other analytical methods for the determination of CBs in water.

Methods	Sorbents	Linear Range (ng L^−1^)	LODs (ng L^−1^)	LOQs (ng L^−1^)	References
SDME/GC-FID	1-Octyl-3-Methylimidazolium hexafluorophosphate	1000–1 × 10^7^	100–500	–	[45]
SDME/GC-MS	Toluene	500–50,000	10–50	40–440	[46]
HSSE/GC-MS	PDMS coated stir bar	10–200	0.4–1.4	1.4–4.7	[47]
SPME/GC-ECD	Nanoporous carbon	10–500 2.5–100	0.10–1.03	–	[48]
HS-SPME/GC-MS	Nano-structured ZnO	0.05–1000	0.01–0.1	–	[49]
SPME/GC-ECD	Nitrogen/Porous biochar	1–1000	0.007–0.079	0.023–0.261	[50]
SPME/GC-MS	CDs/g-C_3_N_4_	0.25–2500	0.002–0.086	0.007–0.288	This work

**Table 3:** Analytical results and recoveries for the determination of CBs in real water samples.

	Analytes	135-TCB	124-TCB	123-TCB	1245-TeCB	1234-TeCB	PeCB	HeCB	PeCNB
1#	Found (ng L^−1^)	ND	ND	1.7	ND	ND	ND	ND	ND
	RSDs (%, *n* = 3)	-	-	0.8	-	-	-	-	-
	Recoveries (%, spiked with 1 ng L^−1^)	105.1	75.5	88.8	94.5	93.8	80.3	101.1	83.4
2#	Found (ng L^−1^)	154.0	ND	11.7	ND	ND	ND	57.6	ND
	RSDs (%, *n* = 3)	11.4	-	13.9	-	-	-	20.6	-
	Recoveries (%, spiked with 10 ng L^−1^)	108.5	105.5	109.1	84.4	84.2	122.3	93.5	75.5
3#	Found (ng L^−1)^	60.4	ND	6.4	ND	ND	ND	ND	ND
	RSDs (%, *n* = 3)	9.9	-	24.6	-	-	-	-	-
	Recoveries (%, spiked with 10 ng L^−1^)	90.8	81.9	83.4	83.1	73.4	76.8	81.2	81.8

## Data Availability

This study presents novel concepts and did not report any data.

## Supplementary Materials

The following supporting information can be downloaded at: https://www.mdpi.com/article/10.3390/nano12030335/s1, Section S1: Operating parameters of GC-MS; Table S1: Retention time and characteristic ions of eight CBs; Table S2: BET and BJH results of as-prepared materials; Figure S1: Reusability of as-prepared SPME fiber.

## References

[1] Liu L., Zhou B., Liu Y., Liu J., Hu L., Tang Y., Wang M. (2022). In-situ regulation of acid sites on Mn-based perovskite@mullite composite for promoting catalytic oxidation of chlorobenzene. J. Colloid. Interf. Sci..

[2] Zhu Q., Yan J., Dai Q., Wu Q., Cai Y., Wu J., Wang X., Zhan W. (2020). Ethylene glycol assisted synthesis of hierarchical Fe-ZSM-5 nanorods assembled microsphere for adsorption Fenton degradation of chlorobenzene. J. Hazard. Mater..

[3] Li F., Wu H., Li Y., He X., Chen L., Zhang Y. (2020). Progress in application of functionalized magnetic nanomaterials for sample pretreatment. Chin. J. Chromatogr..

[4] Arthur C.L., Pawliszyn J. (1990). Solid phase microextraction with thermal desorption using fused silica optical fibers. Anal. Chem..

[5] Reyes-Garces N., Gionfriddo E., Gmez-Rios G.A., Alam M.N., Boyaci E., Bojko B., Singh V., Grandy J., Pawliszyn J. (2018). Advances in solid phase microextraction and perspective on future directions. Anal. Chem..

[6] Yang X., Wang J., Wang W., Zhang S., Wang C., Zhou J., Wang Z. (2019). Solid phase microextraction of polycyclic aromatic hydrocarbons by using an etched stainless-steel fiber coated with a covalent organic framework. Microchim. Acta..

[7] Guo Y., He X., Huang C., Chen H., Lu Q., Zhang L. (2020). Metal–organic framework-derived nitrogen-doped carbon nanotube cages as efficient adsorbents for solid-phase microextraction of polychlorinated biphenyls. Anal. Chim. Acta.

[8] Zhu W., Zhang J., Zhang X., Han L., Qin P., Tian S., Zhou Q., Zhang X., Lu M. (2020). Preparation of Al-doped mesoporous crystalline material-41 as fiber coating material for headspace solid-phase microextraction of polycyclic aromatic hydrocarbons from human urine. J. Chromatogr. A.

[9] Chen L., Huang J., Shi Y., Peng X., Kuang Y., Zhou S., Zheng J., Yang X., Ouyang G. (2022). Polystyrene-based nanospheres with controllable microstructures for exceptional solid phase microextraction of organic pollutants. Chem. Eng. J..

[10] Song X., Wang R., Wang X., Han H., Qiao Z., Sun X., Ji W. (2022). An amine-functionalized olefin-linked covalent organic framework used for the solid-phase microextraction of legacy and emerging per- and polyfluoroalkyl substances in fish. J. Hazard. Mater..

[11] Cheng H., Song Y., Bian Y., Ji R., Wang F., Gu C., Yang X., Ye M., Ouyang G., Jiang X. (2019). Meso-/microporous carbon as an adsorbent for enhanced performance in solid-phase microextraction of chlorobenzenes. Sci. Total Environ..

[12] Ganguly S., Das P., Das T.K., Ghosh S., Das S., Bose M., Mondal M., Das A.K., Das N.C. (2020). Acoustic cavitation assisted destratified clay tactoid reinforced in situ elastomer-mimetic semi-IPN hydrogel for catalytic and bactericidal application. Ultrason. Sonochem..

[13] Das P., Ganguly S., Saha A., Noked M., Margel S., Gedanken A. (2021). Carbon-dots-initiated photopolymerization: An in situ synthetic approach for MXene/Poly(norepinephrine)/copper hybrid and its application for mitigating water pollution. ACS Appl. Mater. Inter..

[14] Shi W., Wang J., Yang S., Lin X., Guo F., Shi J. (2020). Fabrication of a ternary carbon dots/CoO/g-C_3_N_4_ nanocomposite photocatalyst with enhanced visible-light-driven photocatalytic hydrogen production. J. Chem. Technol. Biotechnol..

[15] Dadigala R., Bandi R., Gangapuram B.R., Guttena V. (2017). Carbon dots and Ag nanoparticles decorated g-C3N4 nanosheets for enhanced organic pollutants degradation under sunlight irradiation. J. Photochem. Photobiol. A Chem..

[16] Feng H., Qian Z. (2018). Functional carbonquantum dots: A versatile platform for chemosensing and biosensing. Chem. Rec..

[17] Boakye-Yiadom K.O., Kesse S., Opoku-Damoah Y., Filli M.S., Aquib M., Joelle M.M.B., Farooq M.A., Mavlyanova R., Raza F., Bavi R. (2019). Carbon dots: Applications in bioimaging and theranostics. Int. J. Pharmaceut..

[18] Chen J., Gong Z., Tang W., Row K.H., Qiu H. (2021). Carbon dots in sample preparation and chromatographic separation: Recent advances and future prospects. Trac-Trend. Anal. Chem..

[19] Li Y.-K., Yang T., Chen M.-L., Wang J.-H. (2018). Supported carbon dots serve as high-performance adsorbent for the retention of trace cadmium. Talanta.

[20] Li Y., Zhang X., Wang Y., Lin Y., Zhou J. (2018). Loading controlled magnetic carbon dots for microwave-assisted solid-phase extraction: Preparation, extraction evaluation and applications in environmental aqueous samples. J. Sep. Sci..

[21] Cai T., Zhang H., Rahman A.F.M.M., Shi Y.-P., Qiu H. (2017). Silica grafted with silanized carbon dots as a nano-on-micro packing material with enhanced hydrophilic selectivity. Microchim. Acta.

[22] Cai T., Zhang H., Chen J., Li Z., Qiu H. (2019). Polyethyleneimine-functionalized carbon dots and their precursor co-immobilized on silica for hydrophilic interaction chromatography. J. Chromatogr. A.

[23] Sun Y.-P., Ha W., Chen J., Qi H.-Y., Shi Y.-P. (2016). Advances and applications of graphitic carbon nitride as sorbent in analytical chemistry for sample pretreatment: A review. Trac-Trend. Anal. Chem..

[24] Zhang J., Li W., Zhu W., Qin P., Lu M., Zhang X., Miao Y., Cai Z. (2019). Mesoporous graphitic carbon nitride@NiCo_2_O_4_ nanocomposite as a solid phase microextraction coating for sensitive determination of environmental pollutants in human serum samples. Chem. Commun..

[25] Yang Y., Qin P., Zhang J., Li W., Zhu J., Lu M., Cai Z. (2018). Fabrication of nanoscale graphitic carbon nitride/copper oxide hybrid composites coated solid-phase microextraction fibers coupled with gas chromatography for determination of polycyclic aromatic hydrocarbons. J. Chromatogr. A.

[26] Han L., Yang Y., Zhang J., Guo J., Lu M. (2020). Recent advances in graphitic carbon nitride materials for sample pretreatment. Chin. J. Chromatogr..

[27] Wang Y., Liu X., Liu J., Han B., Hu X., Yang F., Xu Z., Li Y., Jia S., Li Z. (2018). Carbon quantum dot implanted graphite carbon nitride nanotubes: Excellent charge separation and enhanced photocatalytic hydrogen evolution. Angew. Chem. Int. Edit..

[28] Dillip G.R., Sreekanth T.V.M., Joo S.W. (2017). Tailoring the bandgap of N-rich graphitic carbon nitride for enhanced photocatalytic activity. Ceram. Int..

[29] Xu S., Dong P., Qin M., Liu H., Long A., Chen C., Feng S., Wu H. (2021). Core-shell structured Fe_2_O_3_/CeO_2_@MnO_2_ microspheres with abundant surface oxygen for sensitive solid-phase microextraction of polycyclic aromatic hydrocarbons from water. Microchim. Acta.

[30] Xu S., Liu Q., Wang C., Xiao L., Feng S., Li N., Chen C.-P. (2020). Three-dimensional pompon-like Au/ZnO porous microspheres as solid phase microextraction coating for determination of volatile fatty acids from foot odor. Talanta.

[31] Zhang X., Han L., Li M., Qin P., Li D., Zhou Q., Lu M., Cai Z. (2021). Nitrogen-rich carbon nitride as solid-phase microextraction fiber coating for high-efficient pretreatment of polychlorinated biphenyls from environmental samples. J. Chromatogr. A.

[32] Zhang H., Zhao L., Geng F., Guo L.-H., Wan B., Yang Y. (2016). Carbon dots decorated graphitic carbon nitride as an efficient metal-free photocatalyst for phenol degradation. Appl. Catal. B Environ..

[33] Wang K., Wang X., Pan H., Liu Y., Xu S., Cao S. (2018). In situ fabrication of CDs/g-C_3_N_4_ hybrids with enhanced interface connection via calcination of the precursors for photocatalytic H_2_ evolution. Int. J. Hydrogen Energy.

[34] Groenewolt M., Antonietti M. (2005). Synthesis of g-C_3_N_4_ nanoparticles in mesoporous silica host matrices. Adv. Mater..

[35] Li K., Su F.-Y., Zhang W.-D. (2016). Modification of g-C_3_N_4_ nanosheets by carbon quantum dots for highly efficient photocatalytic generation of hydrogen. Appl. Surf. Sci..

[36] Jian X., Liu X., Yang H.-M., Li J.-G., Song X.-L., Dai H.-Y., Liang Z.-H. (2016). Construction of carbon quantum dots/proton-functionalized graphitic carbon nitride nanocomposite via electrostatic self-assembly strategy and its application. Appl. Surf. Sci..

[37] Tripathi A., Narayanan S. (2019). Potassium doped graphitic carbon nitride with extended optical absorbance for solar light driven photocatalysis. Appl. Surf. Sci..

[38] Yan Q., Zhao C., Zhang L., Hou Y., Wang S., Dong P., Lin F., Wang Y. (2019). Facile two-step synthesis of porous carbon nitride with enhanced photocatalytic activity using a soft template. Acs. Sustain. Chem. Eng..

[39] Cui L., Liu Y., Fang X., Yin C., Li S., Sun D., Kang S. (2018). Scalable and clean exfoliation of graphitic carbon nitride in NaClO solution: Enriched surface active sites for enhanced photocatalytic H_2_ evolution. Green Chem..

[40] Feng Z., Huang C., Guo Y., Liu W., Zhang L. (2020). Graphitic carbon nitride derivative with large mesopores as sorbent for solid-phase microextraction of polycyclic aromatic hydrocarbons. Talanta.

[41] Xu L., Huang S., Liu Y., Wei S., Chen G., Gong Z., Ouyang G. (2020). Hollow carbon nanobubbles-coated solid-phase microextraction fibers for the sensitive detection of organic pollutants. Anal. Chim. Acta.

[42] Liu S., Xie L., Zheng J., Jiang R., Zhu F., Luan T., Ouyang G. (2015). Mesoporous TiO2 nanoparticles for highly sensitive solid-phase microextraction of organochlorine pesticides. Anal. Chim. Acta.

[43] Pei M., Shi X., Wu J., Huang X. (2019). Graphene reinforced multiple monolithic fiber solid-phase microextraction of phenoxyacetic acid herbicides in complex samples. Talanta.

[44] Wang Z., Wang F., Zhang R., Wang Z., Du X. (2019). A new strategy for electrochemical fabrication of manganese dioxide coatings based on silica nanoparticles deposited on titanium fibers for selective and highly efficient solid-phase microextraction. New J. Chem..

[45] Zhao F., Lu S., Du W., Zeng B. (2009). Ionic liquid-based headspace single-drop microextraction coupled to gas chromatography for the determination of chlorobenzene derivatives. Microchim. Acta.

[46] Ma X., Ma J. (2017). Determination of trace amounts of chlorobenzenes in water using membrane-supported headspace single-drop microextraction and gas chromatography–mass spectrometry. J. Anal. Chem..

[47] Cacho J.I., Campillo N., Viñas P., Hernández-Córdoba M. (2019). A simple device for headspace sorptive extraction prior to gas chromatography–mass spectrometry analysis. Talanta.

[48] Cheng H., Song Y., Bian Y., Wang F., Ji R., He W., Gu C., Ouyang G., Jiang X. (2017). A nanoporous carbon material coated onto steel wires for solid-phase microextraction of chlorobenzenes prior to their quantitation by gas chromatography. Microchim. Acta.

[49] Ghasemi E., Sillanpää M. (2014). Optimization of headspace solid phase microextraction based on nano-structured ZnO combined with gas chromatography–mass spectrometry for preconcentration and determination of ultra-traces of chlorobenzenes in environmental samples. Talanta.

[50] Ji R., Wu Y., Bian Y., Song Y., Sun Q., Jiang X., Zhang L., Han J., Cheng H. (2021). Nitrogen-doped porous biochar derived from marine algae for efficient solid-phase microextraction of chlorobenzenes from aqueous solution. J. Hazard. Mater..

