# Supplementary material for: Carbon Dot-Decorated Graphite Carbon Nitride Composites for Enhanced Solid-Phase Microextraction of Chlorobenzenes from Water
Source: Nanomaterials (Basel). 2022 Jan 21;12(3):335. doi: 10.3390/nano12030335 (PMC8838722; doi:10.3390/nano12030335)
Supplement: Supplementary file 1 [file nanomaterials-12-00335-s001.zip › nanomaterials-1531991-supplementary.pdf]

nanomaterials MDPI

Supplementary Information

Carbon Dot-Decorated Graphite Carbon Nitride Composites for Enhanced Solid-Phase Microextraction of Chlorobenzenes from Water

Shengrui Xu [1,2,*], Hailin Liu [1], Anying Long [3], Huimin Li [1], Changpo Chen [1], Suling Feng [1,*] and Jing Fan [2]

[1] Key Laboratory of Green Chemical Media and Reactions, Ministry of Education, Collaborative Innovation Center of Henan Province for Green Manufacturing of Fine Chemicals, School of Chemistry and Chemical Engineering, Henan Normal University, Xinxiang 453007, China; LiuHailin20211001@163.com (H.L.); ming1391027849@163.com (H.L.) ; andychen2005@163.com (C.C.)
[2] Henan Key Laboratory for Environmental Pollution Control, Key Laboratory for Yellow River and Huai River Water Environmental Pollution and Control, Ministry of Education, School of Environment, Henan Normal University, Xinxiang 453007, China; fanjing@htu.cn
[3] 113 Geological Brigade, Guizhou Bureau of Geology and Mineral Resources, Liupanshui 553000, China; longying031042@163.com
* Correspondence: xushengrui@126.com (S.X.); slfeng@htu.cn (S.F.)

Section S1. Operating parameters of GC-MS

The parameters and conditions of GC-MS are as follows:

High-purity helium (Beipu Gas Co., Ltd., Xinxiang, China) was used as carrier gas at a flow rate of 1 mL min^{-1}. The injector was operated with splitless mode, and temperature was set at 260 °C. The initial temperature of the column oven was set to 80 °C for 2 min, then increased to 150 °C at a rate of 15 °C min^{-1} and held for 1 min. After that, the oven temperature was increased to 220 °C at 8 °C min^{-1}, and kept for 3 min, finally increased to 280 °C at 20 °C min^{-1} and held for 2 min. The MS was operated with SIM mode with 150 and 230 °C for MS Quad and MS source temperature, respectively. The characteristic ions and retention time of eight CBs are listed in Table S1.

Table S1. Retention time and characteristic ions of eight CBs.

Compounds	Retention (min)	Quantitative ions	Characteristic ions
135-TCB	5.83	184	180、182、184
124-TCB	6.33	184	180、182、184
123-TCB	6.72	184	180、182、184
1235-TeCB	8.10	220	216、214、218、220
1245-TeCB	8.74	220	216、214、218、220
PeCB	10.66	254	24、248、252、254
HeCB	13.25	288	284、286、282、288
PeCNB	14.02	295	237、249、295、214、265

Table S2. BET and BJH results of as-prepared materials.

Materials	S_{BET} / $m^2 g^{-1}$	Pore volume/ $cm^3 g^{-1}$	Average pore diameter / nm
g-C_3N_4	13.34	0.1013	33.7
CDs/g-C_3N_4-3	20.67	0.1078	39.3

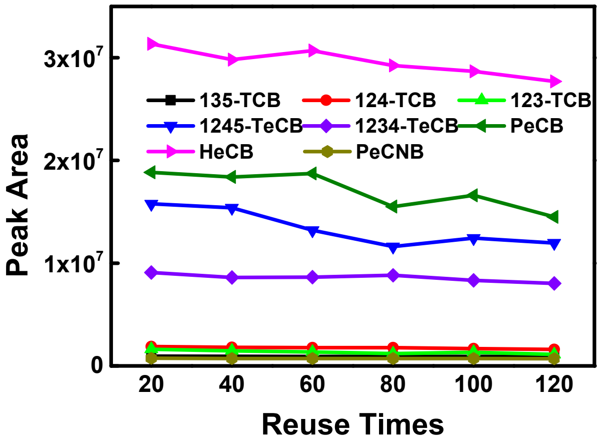

Figure S1. Reusability of as-prepared SPME fiber.
